# Embedding telephone therapy in statutory mental health services: a qualitative, theory-driven analysis

**DOI:** 10.1186/s12888-016-0761-5

**Published:** 2016-03-01

**Authors:** Penny Bee, Karina Lovell, Zerena Airnes, Anna Pruszynska

**Affiliations:** School of Nursing, Midwifery and Social Work, University of Manchester, Manchester, UK; Primary Care Psychological Therapies Service, NHS Tameside and Glossop, Glossop, UK

**Keywords:** Acceptability, Telephone, Cognitive behavioural therapy, Telemedicine, Mental health implementation, Normalisation process theory, Nursing

## Abstract

**Background:**

Telephone-administered cognitive behavioural therapy (T-CBT) has attracted international recognition as a potential means of providing effective psychological treatment whilst simultaneously lowering costs, maximizing service efficiency and improving patient access to care. A lack of rigorous exploration of therapist perspectives means that little is known about professional readiness to adopt such delivery models, or the work that may be involved in ensuring successful implementation.

**Methods:**

This paper reports on a qualitative exploration of professional views of high intensity T-CBT. Semi-structured interviews with 18 UK accredited Cognitive Behavioural Therapists with nursing or allied health backgrounds were collected and analysed according to Normalisation Process Theory, a contemporary and empirically-derived theory of health technology implementation.

**Results:**

Despite increasing research effort seeking to determine the effectiveness of T-CBT, the clinical rationale for its use remains insecure. Professional perceptions of T-CBT as a high risk delivery strategy emerge as a key factor delaying T-CBT routinisation in practice. T-CBT champions draw on experiential knowledge to demonstrate that remote services can add value, a key factor being the recognition that telephone-mediated services can provide viable access for hard to reach populations. T-CBT uptake will be facilitated by i) the modification of existing protocols to address new methods of exchanging information and data, and (ii) greater clarification of the reach and span of telephone therapies, including the most appropriate division of labour across different service levels and settings.

**Conclusions:**

The integration and normalisation of high intensity T-CBT into mental health services demands greater recognition and redress of the existing socio-technical matrices within which nursing and allied health practitioners work. The future spread of higher intensity T-CBT is contingent upon the willingness of service managers to support staff in the delivery and governance of non-face-to-face therapy models. Clear delineation of the role and scope of T-CBT and the extent to which it will extend or replace existing provision is required.

## Background

Today, rapid developments in communications technologies are enabling healthcare providers to implement new ways of consulting with patients and delivering complex interventions. Contemporary mental healthcare offers one such example, with increasing attention being focused on the development and evaluation of remote psychotherapies [1]. Potential synergies between empirically grounded psychological techniques and a ubiquitous communication technology capable of mediating collaborative problem solving exercises have led to the championing of telephone-supported guided self-help as a pragmatic solution to rising demand and inequitable access across different geographical regions and patient groups [2, 3]. These developments complement a contemporary and philosophical shift towards improving the quality of mental health care, a policy initiative that demands the provision of greater opportunities for patient preference and choice [4].

At the time of writing, lower intensity telephone interventions, such as guided self-help (GSH) constituted an integral part of the ‘stepped care’ model adopted by the UK’s Increasing Access to Psychological Therapies (IAPT) initiative. Predicated on a combination of economic and research evidence, this system of healthcare provision is designed to maximise service efficiency by delivering the most effective yet least resource intensive treatment first [5]. Brief telephone interventions are advocated as a core part of the stepped care model and feature in national IAPT training manuals [6].

The adoption of remote psychotherapies into higher tiers of the stepped care model has been slower. International research efforts have explored the feasibility, efficacy and acceptability of high intensity telephone CBT (T-CBT) across a range of disorders and settings . Effect sizes vary across studies and populations [7–11]. Meta-analysis of T-CBT for depression [9] demonstrates significant reductions in post-treatment symptoms when compared to pre-treatment scores (standard mean gain 0.81; 12 RCTs). Smaller effect sizes are reported when T-CBT is compared to control conditions (standard mean difference 0.26, 10 RCTs) but heterogeneity in comparators limits meaningful interpretation.

Few trials have measured client satisfaction. Those that have report similar or greater satisfaction to comparable interventions delivered face-to- face [1]. Such quantitative assessments remain limited in their ability to explore important process factors, including the relative contribution of individual values and expectations in determining therapy uptake and service preference. Although qualitative analyses of users’ views of T-CBT are available [12], empirical investigations of the professional perspective are sparse.

Existing literature documents the challenges encountered in the implementation of telemedicine more generally, highlighting potential disparity between political interest in telemedicine and the extent to which nursing and allied health professionals may be willing to engage with such services on the ground [13]. Studies of remote psychiatric assessments confirm that embedding technological innovation into statutory healthcare will demand multiple changes to the structure and delivery of mental health services [14], with the likelihood that the normalization of a new health technology will be mediated both by the properties of the technology itself and the entrenched sociological orientation of its key stakeholders.

Studies focused on one-off remote consultations [15] provide a preliminary framework for the study of remote psychotherapy provision, but are unable to elucidate the specific constraints or enablers facing their longer term routinisation [16, 17]. Humanistic psychotherapists remain steadfast in emphasizing the importance of the therapeutic relationship as a core mechanism of action in therapy, and in viewing empathic listening [18], evolving from both verbal and non-verbal cues, as an essential component of the therapeutic encounter [19]. In demonstrating the clinical effectiveness of higher intensity T-CBT, research evidence has challenged both of these concepts. Higher intensity T-CBT thus represents a contemporary example of a ‘technological innovation’ in mental healthcare and a salient means by which to study health professionals’ negotiation of a new delivery model in practice.

Our aim was to explore cognitive behavioural therapists’ narratives around T- CBT, with a view to identifying current and potential influences on its uptake and implementation in statutory mental health services.

### Theoretical perspective

The routinisation of health technologies is widely recognised as a complex process. Current conceptualisations evolve out of several diverse disciplines including knowledge transfer, evidence-based medicine and quality improvement initiatives [20]. Concomitantly, the processes of technology uptake and spread form the basis of multiple sociological theories. Supported by a robust evidence base, early diffusion theories advocate the transmission of innovations via social networks (e.g. Diffusion of Innovations Theory [21]) or via new or existing relationships between artifacts and people (e.g. Actor Network Theory [22]). More recently Normalisation Process Theory (NPT) has emerged [23, 24].

NPT is directly concerned with the tasks involved in innovation implementation. Rather than seeking to explain processes of spontaneous adoption, NPT focuses on ‘the social processes that play out in response to a deliberately initiated and institutionally sanctioned decision to adopt technology in a specific setting.’ [23]. In doing so, it identifies a set of explanatory mechanisms that empirical investigation [13, 24] has identified as key to technology normalisation in practice.

**Fig. 1:**
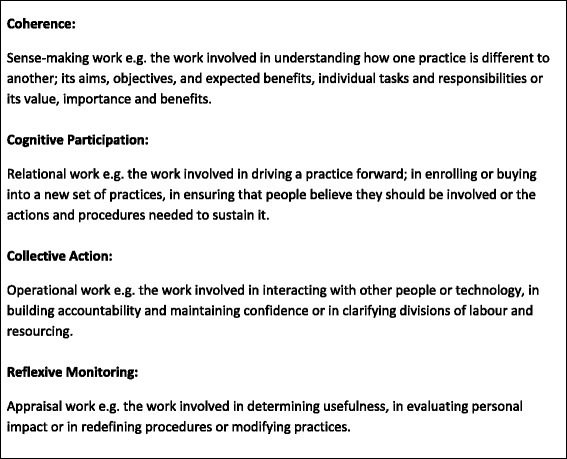
Core constructs of normalisation process theory

NPT consists of four main constructs: Coherence (the work that people do to understand and make sense of a complex intervention); Cognitive Participation (the manner by which they engage with the intervention); Collective Action (the way in which they enact it and Reflexive Monitoring (the work that they do to appraise its effects). Greater details regarding the scope and definition of these four constructs is provided in Fig. 1.

## Methods

### Ethics, consent and permissions

In order to access a nationally diverse sample of practitioners, study flyers and consent to contact forms were handed out to attendees at the British Association for Behavioural and Cognitive Psychotherapies (BABCP) Annual Conference. Participation in the study was entirely voluntary and not associated with any monetary or professional reward. Participants who did not provide their contact details were not followed up and were not disadvantaged in any way. Conference attendance was not affected by study participation.

Consent to contact was assumed on the basis of potential participants providing their contact information. Detailed study information sheets were posted to all individuals expressing an interest in the study and consenting to further contact. Study information sheets were sent at least 48 hours prior to informed written consent for the interviews being taken. Interview consent forms were signed by participants and returned to the research team by post or in person depending upon location. Research team members were available by email or telephone to answer questions. Interviews were not scheduled until written consent was received. The study and the above procedures were approved by the University Research Ethics Committee, University of Derby, UK (UREC REF: AP 11/10).

### Participants and recruitment

Participants were recruited via convenience sampling from a national pool of nurses and allied health professionals who had qualified and were practicing as cognitive behavioural therapists. Inclusion criteria required participants to meet minimum criteria for BABCP accreditation i.e. a minimum of 200 hours supervised assessment and cognitive behavioural therapy during training. All those who expressed an interest in the study and met study eligibility requirements were invited to interview.

Thirty eight flyers were distributed and 29 individuals expressed an interest in the study and consented to researcher contact. The reasons why nine people did not want to be contacted were not collected. Two of the 29 expressing an interest in the study did not meet study inclusion criteria. Five could not be reached using the contact details they provided, and four did not complete their interviews. Eighteen individuals (9 males, 9 females) therefore participated in interviews. Nine were registered mental health nurses, the remainder of the sample being comprised of allied health and social care professionals. All participants had experience of delivering CBT in statutory health services (*n* = 12), education (*n* = 4), voluntary (*n* = 2) and/or private practice (*n* = 2). Two worked in dual settings and therefore total numbers exceed sample size. Professional experience ranged from 1 to 20 years (mean (SD): 7.6 (5.4) years). Twelve (66 %) had prior experience of delivering telephone CBT (Table 1).

**Table 1:** Participant sample characteristics

ID	Gender	Accreditation years^a^	Profession	Employing organisation	T-CBT experience
1	F	1	Registered mental health nurse	NHS	Occasional session
2	F	3	Counselling	NHS; Private	None
3	M	16	Registered nurse and mental health nurse	Education; Private	Full therapy
4	F	1	Registered mental health nurse	NHS	Occasional session
5	M	3	Occupational therapist	NHS	None
6	F	7	Registered mental health nurse and LD nurse	NHS	Occasional session
7	M	15	Registered mental health nurse	Voluntary sector	Full therapy
8	M	20	Registered mental health nurse	Voluntary sector	Full therapy
9	F	5	Counselling	Education	None
10	F	3	Occupational therapist	NHS	Full therapy
11	F	10	Clinical psychologist	NHS	None
12	M	13	Clinical psychologist	NHS	None
13	M	1	Graduate mental health worker	NHS	None
14	F	1	Social worker	NHS	Occasional session
15	M	7	Registered mental health nurse	Education	Full therapy
16	M	13	Registered mental health nurse	NHS	Full therapy
17	F	11	Registered mental health nurse	NHS	Occasional session
18	M	7	Health psychologist	Education	Occasional session

^a^since meeting BABCP minimum standards; *LD* Learning disabilities

### Procedures

Data collection was undertaken by one female researcher trained in qualitative methods (AP). At the time of data collection, AP was a qualified Cognitive Behavioural Therapist completing an MSc in CBT. The interviewer did not have any prior experience of delivering T-CBT. She was not known by study participants and professional status was not disclosed at time of interview. Participants were informed that the study was being conducted in part fulfilment of an academic qualification. With the exception of two participants who requested face-to-face interviews at home, all data collection was conducted via the telephone to facilitate participation over a diverse geographical area. No other individuals were present at the time of the interview.

Interviews were conducted using inductive questioning driven by a semi-structured schedule that was devised and piloted by the research team. Interview duration ranged from 39 to 62 min. Interviews were audiotaped and transcribed verbatim. Participants were sent copies of their transcripts for editing and correction purposes. No study withdrawals occurred and no changes to transcript content were required. Field notes were not systematically collected and did not contribute to data analysis.

### Analysis

Data underwent a thematic analysis informed by Normalisation Process theory (NPT). Data were managed in MS-Word 2007 and independently analysed by PB & ZA. Data analysis took place in two phases to avoid forcing data into categories predetermined by the NPT framework [25]. Firstly, thematic analysis was conducted by ZA and independently verified by PB. Emergent themes were coded using a method of constant comparison [26] comparing, classifying and refining codes across interviews until no new themes emerged. The distribution of codes was recorded and any data falling outside of the coding frame was re-examined to determine if important concepts were being missed.

**Fig. 2:**
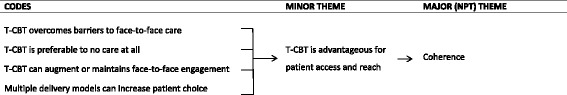
Example extract from the coding tree

In the second phase of the analysis, emergent themes (and constituent codes) were mapped to the NPT framework checking for fit (Fig. 2). Mapping was carried out independently by ZA & PB with discrepancies and/or differences in insight resolved via discussion with a third member of the team (KL). Participant checking of the data coding process was not performed.

## Results

All of the emergent themes identified in the first phase of our analysis mapped onto the NPT framework and no codes were deemed fall outside of its scope (Table 2). We thus structure the presentation of our results around its four key constructs: Coherence, Cognitive Participation, Collective Action and Reflexive Monitoring. Participants are assigned a number rather than a name or pseudonym within the text. Gender and length of professional experience are provided.

**Table 2:** Core constructs of normalisation process theory (Major themes) and study findings (Minor themes)

NPT construct	Emergent study themes
Coherence:	• T-CBT alters practitioner-client communication.
	• T-CBT challenges risk management.
	• T-CBT challenges collaboration.
	• T-CBT may be more limited in content.
	• T-CBT delivery demands different skills.
	• Client diagnosis/case complexity may limit T-CBT utility.
	• T-CBT is advantageous for patient access and reach.
Cognitive Participation:	• T-CBT is a macro and meso level directive.
	• Front line support for T-CBT may be lacking.
	• T-CBT is enabled by professional autonomy.
	• T-CBT is aligned with service efficiency.
	• T-CBT acceptability is influenced by organisational culture.
Collective Action:	• Confidence in T-CBT requires a mixed delivery model.
	• T-CBT is delivered within a risk-minimisation framework.
	• T-CBT implementation requires increased resourcing.
	• T-CBT requires local protocol and policy development.
Reflexive Monitoring:	• Local T-CBT champions exist.
	• T-CBT supporters draw on experiential learning.
	• T-CBT is acceptable in practice.
	• T-CBT has proven client gains.
	• Technical support will enhance information sharing.
	• T-CBT requires dedicated training.

### Coherence: making sense of T-CBT

The inclusion of coherence as a core construct of NPT acknowledges that the introduction of any new health technology will demand the generation of a shared set of ideas regarding its characteristics, utility and meaning [23]. For participants in the current study, the incorporation of T-CBT into practice was conceptualized predominantly as a process of altered communication, with subsequent impact on its therapeutic management and enactment. Irrespective of experience, the vast majority of therapists voiced concerns for patient safety, a conjectural limitation that most commonly arose from the perceived challenges of managing distress and suicidal ideation at a distance:

*“If somebody becomes distressed and starts speaking about self-harm or you know suicidal plans, you know you’ve got them in a safe environment, you can bring them or the services in. Obviously you are a support person for them there at that time. If they’re at home and become distressed and for example hang up the phone it’s much harder to get the right help there at that time.”*

(Participant 3, Male, CBT accredited 16 years)

Less frequently, professional discourse extended beyond risk management to identify other potential constraints on their therapeutic practice. When describing the specific tasks and responsibilities of an effective therapist, participants’ points of reference remained firmly fixed on the face-to-face encounter and its ability to facilitate the exchange of visual data:

*“I think for me the other advantage is to be able to collaboratively formulate. Although I appreciate that over the phone you can guide somebody, so on and so forth, you can’t actually see what they’re doing, exactly how they’re formulating that.”*

(Participant 5, Male, CBT accredited 3 years)

Conceptual differences between telephone and face-to-face delivery were thus rendered largely in terms of their technical properties and the perceived suitability of the two different types of communication for the tasks in hand. Although rarely substantiated by evidence, accredited therapists portrayed an innate cautiousness towards T-CBT that was fuelled by expectations of miscommunication, therapy rupture and client disengagement. This shared dialogue served to limit the role and scope of T-CBT, confining its potential contribution to the periphery of normal practice.

Despite achieving consensus on the technical challenges of remote delivery, contradiction in therapists’ viewpoints showed that full agreement on the objectives and benefits of T-CBT was still to be reached. Several practitioners deliberated on the diverse nature of mental health difficulties, upholding this as the primary reason why T-CBT could never be considered a ‘cure-all’ remedy for service demand. The availability of audio rather than visual data was frequently taken to denote a more limited therapeutic platform, permitting professionals to intervene only with ‘less complex’ cases or provide follow up to ‘more predictable’ clients:

*“Maybe [T-CBT] could be used for somebody who has an anxiety or depression disorder, a common mental health problem, some-one who didn’t have complicated things like childhood trauma, co-morbid personality issues or psychosis, or something like that.”*

(Participant 1, Female, CBT accredited 1 year)

Differentiation between the different remits and challenges posed by telephone and face to face therapies gave rise to a further distinction in the skills required to facilitate the two models. Rather than the limited technological functions of the communication medium demanding additional training however, it became apparent that therapists actually conceived telephone delivery as a lesser form of therapy and thus, as an intervention only sanctioned for delivery by ‘paraprofessionals.’ This demarcation served to preserve the need for conventional therapy and provided one possible route through which T-CBT antagonists could negotiate technological innovation without challenging their own roles and status:

*“I’m not denying the fact that some cognitive behavioural therapy has been delivered over the phone but the majority of it is guided self-help based on cognitive behavioural principles…. basically coaching people through using materials, signposting and so on. It’s not CBT as we know it.”*

(Participant 12, Male, CBT accredited 13 years)

As each participant worked to make sense of a new delivery model, they strived to find some value in T-CBT. Expressions of enthusiasm were for the most part context-limited and focused on the benefits for service users who, through no fault of their own, were unable to access traditional face to face appointments:

*“Reducing physical demands upon people, especially those with physical health problems. Sometimes they have great difficulties attending sessions and it would be nice to be able to increase access to those groups…offer them treatment over the telephone.”*

(Participant 9, Female, CBT accredited 5 years)

Within this context of increasing access for hard to reach groups, there were three main ways in which professional support for T-CBT was framed: i) by benchmarking a potentially sub-optimal (i.e. remote) service against otherwise absent care; ii) by appealing to professional constructs of patient-centred services and choice or iii) by conceptualising T-CBT as a complementary, non-mandatory adjunct to standard delivery models. User access difficulties were typically conceived as externally driven, unavoidable forces and thus legitimate justifications for adapting standard therapeutic procedures. The internalisation of T-CBT thus appeared to be expedited by situations that aligned most readily with professionals’ perspectives of therapy as a context–bounded interaction, and, most importantly, with their own concepts of responsibility service providers’ responsibility to patient care [27].

### Cognitive partcipation: building a T-CBT community

According to NPT, new applications of health technologies require the instigation of new or adapted models of normative conduct. As such, their routinisation is governed by factors that either promote or inhibit people’s participation at both micro and macro levels.

Participants in the current study acknowledged that the integration of T-CBT into statutory mental health services had already begun, but posited that it was, for the most part, the result of a top-down organisational directive rather than a bottom-up need for change. A common perception was that T-CBT was a innovation promising efficiency gains at a macro level, with meso-level managers assuming responsibility for its instigation and use. The net effect was a sense of mistrust of the political origins of T-CBT and of the potential risks that this more innovative delivery model may generate:

*“Within the political climate at the moment there is clear emphasis on making cuts and looking at the cheapest way to do anything. I think there’s a real danger of people grabbing hold of concepts and ideas purely and simply on the financial implication. There may be some evidence that supports that but that will be at the entire risk of what’s there.”*

(Participant 5, Male, CBT accredited 3 years)

Service setting rather than length of professional experience emerged as a potentially important influence on T-CBT enrolment. For practitioners working in private practice or charity contexts, professional autonomy and service efficiency were upheld as two particularly important issues and ones that aligned more readily with the philosophy of the host organisation. The goal of cognitive participation, or buy-in, thus appeared closely related to the social matrices within which individual practitioners worked. Legitimisation of T-CBT was more easily achieved when proponents acknowledged a genuine need for cost savings and/or recognised the benefits of flexible working across both micro and macro levels:

*“There’s no need to travel so you’re going to make it cost effective. Therapists can stay in their pyjamas, can be at home or wherever they want to be. It would allow you to perhaps get more done in the day because you haven’t got the travelling or even the meeting and greeting in the room.” upheld*

(Participant 2, Female, CBT accredited 3 years)

### Collective action: putting T-CBT into practice

Collective action refers to the work that people do to relate to each other and a new health technology in an everyday setting. Having originally conceived T-CBT as a peripheral adjunct to traditional service provision, some of its biggest opponents faced important conceptual barriers to its enactment. Negotiating these barriers was complex work that involved rethinking existing and entrenched patterns of professional behaviour.

A key determinant of therapy success was perceived to be patient engagement, and by implication, the instigation of a strong therapeutic alliance. For many participants therefore, T-CBT was only viable if traditional face to face assessments and introductions remained. This stance was particularly apparent among statutory service employees, who alluded to a need to constrain practice innovation for the purposes of risk minimisation. Building accountability and maintaining confidence in telephone delivered therapy thus demanded a synergistic approach that mixed and optimised the benefits of different delivery models:

*“Only once you’ve established that understanding and you’ve got that assessment process; face to face, only then would I be comfortable using telephone CBT, only under those circumstances.”*

(Participant 12, Male, CBT accredited 13 years)

T-CBT was thus frequently reported to increase rather than reduce the complexity of service provision and necessitate the augmentation rather than the replacement of existing service structures. Necessary support was posited to include the retention of physical infrastructure (i.e. consulting rooms) alongside increased administrative and human resourcing. Pervasive barriers to the frontline implementation of T-CBT were often evident and included a lack of defined processes and dedicated service protocols for effective referral and therapeutic enactment:

*“With regards to phone CBT … looking at more to do with suitability criteria. I think it might be useful to…to look at, to know, what would make one person more suitable for CBT over the telephone [rather] than somebody who could benefit from being in the room.”*

(Participant 18, Male, CBT accredited 7 years)

### Reflexive monitoring: appraising the value of T-CBT

Participants with direct experience of T-CBT recounted the work that they had undertaken in order to understand the ways in which this new delivery model was likely to affect their work and the ways in which T-CBT practices could best be operationalised in practice.

As participants began to explore their first-hand experiences of remote psychotherapy, it became clear that the sense-making work that they had undertaken as individuals had not always remained consistent with the more cautious parameters set at a communal level. Small pockets of enthusiasm for T-CBT emerged and, by implication, the existence and potential power of local practice-based champions:

*“There’s no reason why you can’t engage somebody and develop an alliance over the telephone…there is no disorder that I can think of that wouldn’t be prepared to do telephone treatment with.”*

(Participant 7, Male, CBT accredited 15 years)

With the exception of two therapists whose had adopted telephone services from the outset, study participants described how their own use of T-CBT had often occurred in an adhoc fashion, usually in response to external conditions that threatened therapeutic rupture under more traditional models:

*“In terms of continuity erm… I tend to try and be flexible and provide a session instead of face to face because of the fact that it can encourage them to keep going rather than have people miss. The access often… the phone’s much easier than people getting in because of transport problems, weather, things like that.”*

(Participant 4, Female, CBT accredited 1 year)

Communal appraisal of the benefits of T-CBT was thus typically shaped by reactive rather than proactive forces, and the significance of scientific evidence regarding the clinical effectiveness of TCBT downplayed in preference for experiential learning. Direct exposure to T-CBT had allowed a minority of therapists to explore the true workability of the intervention, enabling them to better negotiate its technical features and build their confidence in its outcomes and safety. Among these participants, remote communication media were much more likely to be championed for their anti-stigmatising properties and their ability to improve access, enhance client disclosure and strengthen, rather than weaken, the therapeutic alliance. Strong support for remote therapy provision was vocalised by these individuals who, having started to feel more confident with T-CBT services began to question the long professed advantages of face-to-face appointments:

*“If they were reluctant to go and see somebody, I would definitely be offering it as an alternative. It’s more acceptable than going to see a therapist, having a chat with somebody over the phone.”*

(Participant 10, Female, CBT accredited 3 years)

As attention shifted from conventional ideologies of psychotherapy to the possible re-shaping of professional behaviours in practice, efforts to demarcate appropriate roles for T-CBT were replaced with attempts to refine the skills required for this unique delivery model. Multiple solutions to a lack of visual data were proposed including the possibility of emailing information to a client and/or the concurrent adoption of information systems capable of sharing client and practitioner resources across time and space:

*“You’d have to set up .. answer phones and mail boxes and stuff like that whereby they could contact you easily and effectively, rather than always leaving a message. So if the telephone becomes the main forum for therapeutic practice you’d have to invest in that, ensure it provides a full service rather than just a piecemeal one.”*

(Participant 14, Female, CBT accredited 1 year)

A recurring and important theme was the notion that the longer term normalisation of T-CBT will invariably demand dedicated training and support. This appeal was made by both lesser and more experienced therapists and was posited to serve multiple goals. Firstly, it conferred direct and individual benefit, enhancing the skills and confidence of those tasked with delivering T-CBT services. Secondly, it offered communal gain, increasing the likelihood that research evidence would be potentially legitimised by professional support from practice. Thirdly, it channeled peer evaluation, ensuring continuity in reflexive monitoring, feedback for future service configuration and further development of service and human infrastructure.

## Discussion

This study of a contemporary mental health innovation shows that the normalisation of T-CBT is mediated both by the properties of the technology itself and the entrenched sociological orientation of its human actors. Theoretical analysis informed by NPT has elucidated the breadth of ways in which the professional therapeutic community is currently engaged in the negotiation and reworking of relational conventions, the parallel enactment and subversion of intended practices and the projection of roles and resourcing of new technologies in the future. Interdisciplinary exploration of healthcare innovation theories has previously conceptualised the identities of those delivering novel interventions as products of sociotechnical interactions partially influenced by macro structures and policy [20]. Whether or not change occurs in the desired way is thus influenced by multiple (and often unpredictable) interactions that arise in specific contexts or service settings.

The repeated failings of health technologies to become embedded in practice have prompted a critical sociological perspective focused on the need to understand implementation resistance [28–30]. Historically, the predominant approach has been to view innovation through a lens of spontaneous adoption with theories of diffusion predicated on assumptions of humans as rational actors [21, 22]. Although not without merit, these perspectives are unlikely to acknowledge the complexity of practitioner experience under pre-meditated (policy-driven) initiatives, and thus potentially ignore key implications for the design and delivery of services under these conditions. Thus, despite research and policy developing a mature discourse on the effectiveness and cost effectiveness of T-CBT [7–11], the perspectives of individuals delivering this intervention have previously evaded attention.

By using qualitative methods to explore the perspectives of accredited therapists, we have been able to reflect upon a range of important factors relevant to the use and future spread of T-CBT. We have shown that an insecure clinical rationale, exacerbated by perceptions of a potentially high risk delivery model, is heavily implicated in the delayed embedding of telephone-mediated services. Although the promise of enhanced service efficiency has captured the attention of policy makers and commissioners, professional identities appear heavily influential in determining the ‘front line’ desirability of T-CBT from the perspective of mental health practitioners. These findings resonate closely with other studies that have highlighted an underlying resistance to telehealth; one that is frequently attributed to professional conservatism regarding the authenticity and morality of geographically dispersed care [13, 16, 17].

The current study demonstrates how meaning-generating actors (in this case accredited cognitive behavioural therapists) can work both individually and collectively to influence the acceptance or subversion of evidence based initiatives. Any complex intervention seeking normalisation in practice requires stakeholders to create a level of stability, derived from a common understanding of its purpose and advantage. Our findings show that current perceptions of T-CBT are rarely focused on how the therapeutic alliance may be liberated by communication technology so much as on how professional values may be denigrated by new service developments. As such our study serves as an important signpost for mental health service commissioning, highlighting an urgent need for policy, research and practice to acknowledge the dynamics and behaviours inherent in T-CBT implementation.

Our study explored the views of experienced, accredited cognitive behavioural therapists with a view to identifying current and potential influences on higher intensity T-CBT in statutory mental health services. Brief telephone interventions are already an integral feature of stepped care services, and often act as the predominant or sole delivery method for lower intensity intervention. Potential for synergistic learning between the two treatment tiers therefore exists. Explicit recognition must nonetheless be given to the different clinical and risk profiles likely to be seen in higher intensity services, and to the specific challenges involved in negotiating practice change. Understanding the socio-political identities of T-CBT and the likely impact of these on its normalisation are likely be a vital element of this process. Only when individual and group appraisals align readily with perceptions of professional roles and identities are the benefits of the new service more likely to outweigh the commitment required to adopt a remote service model. As such, the spread and enactment of T-CBT may be facilitated in situations that complement rather than contest the stability and normality of existing conduct.

Patients’ appreciation of remote therapy provision have previously been represented in different ways spanning both quantitative ratings [1, 31] and in-depth qualitative exploration [12]. Emphasis of the perceived benefits of T-CBT from this perspective may ultimately appeal to embedded constructs of professional responsibilities to enhance client wellbeing and choice, and thus a potentially important mechanism by which the communal specification of remote psychological therapy can be strengthened. The acceptance of this evidence will undoubtedly consolidate the argument that users of mental health services advocate flexible and remote links to care. Whether or not this ‘bottom up’ approach is sufficient to outweigh professional opposition is less clear.

A central tenet of NPT is that new services are rarely provided in a vacuum. Instigating new ways of working thus demands a wealth of invisible as well as visible work in order to assimilate old and new approaches and to establish a clear method through which new technologies are resourced and enacted. T-CBT in particular appears to raise some important questions about the way in which services might best be configured to accommodate new forms of practice. At the individual level, high intensity practitioners need to make adjustments to their own communication styles, making sure that traditional face to face practices are adapted to accommodate a lack of visual data, ensure therapeutic interaction and achieve the best possible outcomes for distally located users.

According to NPT, a health technology must always be specified in terms that are understandable to, and shared by the people who engage with it [23, 24]. Accepting the findings of the current study as a contemporary indicator of professionals’ sense- making behaviour intimates that communal specification of T-CBT is currently problematic. Drivers of remote therapy take many forms, encompassing the merits of enhanced user access as well as misgivings related to the need to improve service efficiency in the face of potential risk concerns. On top of this are layered differences in the relative maturities of the underpinning evidence base for different disorders and differences in service configurations that lead to variability in professionals’ understandings of their roles.

Multiple factors promoting the successful implementation of T-CBT were identified including: (i) a perception of the relative worth of remote access for hard to reach service users; (ii) a willingness to learn from others and to champion T-CBT in practice; (iii) current or future opportunities to make modifications to technical and therapeutic protocols including new ways of exchanging information and data, and (iv) greater clarification of the reach and span of telephone therapies including the most appropriate division of labour and responsibility across different services and intervention intensities. As long as ambiguity remains over the most appropriate contexts and audiences for T-CBT, uncertainty will exist in workload allocation.

The model of coherence identified by the current study has significance not only in terms of how a new technology is given meaning, but also in demonstrating the multiple ways in which individuals can respond and react to innovation. Within our study, greatest support for T-CBT came from individuals who had already encountered this delivery model, often within non-statutory practice. By identifying such ‘socio-technical champions’, and shedding light on a potential mismatch between individual and communal appraisals of T-CBT, our data suggest that the implementation of telephone therapy continues to hold promise. Encouraging the adoption of remote psychotherapies through the provision of appropriate and specialised training may thus be a more immediate way of harnessing professional capacity to modify experience and a novel mechanism through which to embed T-CBT in practice.

This novel exploration of cognitive behavioural therapists’ views provides critical insight into some of the practices and processes of professional experience in a socio-technical context. Concomitantly, it is subject to many limitations inherent in qualitative research, especially with respect to generalisability. Stratified sampling of interviewees was not possible and as such our sample includes all those individuals who were approached and who consented to an interview. Although sample size was ultimately set by the number of consenting participants, data saturation was achieved. The involvement of participants from different sectors and backgrounds maximizes heterogeneity in professional experience.

Recruiting nurses and allied health professionals from a pool of national conference attendees raises the potential for bias, since it may be posited that conference attendees may be more engaged in evidence based practice and knowledge exchange than non-attendees. Equally, it may be argued that any resistance to new health technologies that is expressed by this forward thinking sample is also likely to be expressed by others and is thus of crucial importance to both policy and practice. Predicting the local spread of T-CBT requires more detailed information on service contexts and working conditions, and the likely ration of conducive to impeding factors. Quantitative or mixed methods research capable of evaluating different implementation models would offer an additional perspective.

The current study was primarily one of institutionally-sanctioned rather than spontaneous (naturally occurring) change. The inclusion of nurse practitioners with more limited exposure to T-CBT raises the possibility that perceived levels of resistance to the technology were elevated by the context in which it was discussed. Arguably, it also enables the identification of early implementation barriers and thus examination of a greater breadth of process from research dissemination to knowledge utilisation.

The recruitment of a diverse group of nursing and allied health professionals from different service settings and geographical contexts lead to conclusions and implementation recommendations that are broad in their relevance. Simultaneously however, some of the findings offer insights into the adoption of mental health technologies at meso and macro levels and thus may not be limited solely to telephone interventions. Parallel and future developments in mental health care are beginning to demand the adoption of other innovative health technologies such as smartphone therapies, the routinisation of which may encounter similar if not greater barriers to those reported here. Innovation can present a direct and systemic threat to organisational norms [32] and will require a conscious and determined approach to service adaptation.

We were unable to obtain ethnographic observation data on organisational interactions, and as such our analysis remains exploratory. A combination of deductive and inductive approaches has allowed us to go beyond the discourses dominating mental health research, to elicit and articulate potentially important socio-medical and socio-technical influences on T-CBT stabilisation. Whilst policy stances are premised on scientific evidence and/or the need for efficient investment decisions [33], local behaviours are much more likely to be founded on a mix of rhetoric, boundary-drawing and individual specification [14]. We thus broaden the evidence base for T-CBT, to include a conceptual assessment of the social processes through which such outcomes may be realised.

## Conclusion

Substantial debate continues regarding the safety and optimal reach of telephone services, including the appropriate division of labour across different therapeutic intensities and delivery contexts. Normalisation of high intensity T-CBT demands much greater recognition and redress of the socio-technical matrices within which mental health nurses and allied health professionals work.
